# Implementing universal Lynch syndrome screening (IMPULSS): protocol for a multi-site study to identify strategies to implement, adapt, and sustain genomic medicine programs in different organizational contexts

**DOI:** 10.1186/s12913-018-3636-2

**Published:** 2018-10-30

**Authors:** Alanna Kulchak Rahm, Deborah Cragun, Jessica Ezzell Hunter, Mara M. Epstein, Jan Lowery, Christine Y. Lu, Pamala A. Pawloski, Ravi N. Sharaf, Su-Ying Liang, Andrea N. Burnett-Hartman, James M. Gudgeon, Jing Hao, Susan Snyder, Radhika Gogoi, Ilene Ladd, Marc S. Williams

**Affiliations:** 1 Geisinger Genomic Medicine Institute, 100 N. Academy Ave, Danville, PA 17822 USA; 2 University of South Florida, 3720 Spectrum Blvd, Suite 304, Tampa, FL 33612 USA; 3 Center for Health Research, Kaiser Permanente Northwest, 3800 N. Interstate Ave, Portland, OR 97202 USA; 4 Department of Medicine and the Meyers Primary Care Institute, University of Massachusetts Medical School, 365 Plantation St. Biotech 1, Suite 100, Worcester, MA 01605 USA; 5 Colorado Center for Personalized Medicine, University of Colorado, Aurora, CO 80045 USA; 6 Department of Population Medicine, Harvard Medical School and Harvard Pilgrim Health Care Institute, Boston, USA; 7 HealthPartners Institute, Bloomington, MN USA; 8 Division of Gastroenterology, Department of Medicine, Department of Healthcare Policy and Research, Weill Cornell Medicine, New York, NY USA; 9 Palo Alto Medical Foundation Research Institute, 795 El Camino Real, Palo Alto, CA 94301 USA; 10 Kaiser Permanente Colorado, Institute for Health Research, 2550 S. Parker Rd., Ste 200, Aurora, CO 80014 USA; 11 Intermountain Healthcare, Precision Genomics, IMC campus, Bldg. 2, Suite 610, 5121 S. Cottonwood Street, Murray, UT 84107 USA; 12 Geisinger Department of Epidemiology and Health Services Research 100 N, Academy Ave Danville, Mahoning Township, PA 17822 USA

**Keywords:** Lynch syndrome, Implementation, Qualitative comparative analysis (QCA), Consolidated framework for implementation research (CFIR), Precision medicine, Universal screening, Colorectal Cancer, Economic evaluation, Decision analytic modeling, Configurational comparative method

## Abstract

**Background:**

Systematic screening of all colorectal tumors for Lynch Syndrome (LS) has been recommended since 2009. Currently, implementation of LS screening in healthcare systems remains variable, likely because LS screening involves the complex coordination of multiple departments and individuals across the healthcare system. Our specific aims are to (1) describe variation in LS screening implementation across multiple healthcare systems; (2) identify conditions associated with both practice variation and optimal implementation; (3) determine the relative effectiveness, efficiency, and costs of different LS screening protocols by healthcare system; and (4) develop and test in a real-world setting an organizational toolkit for LS screening program implementation and improvement. This toolkit will promote effective implementation of LS screening in various complex health systems.

**Methods:**

This study includes eight healthcare systems with 22 clinical sites at varied stages of implementing LS screening programs. Guided by the Consolidated Framework for Implementation Research (CFIR), we will conduct in-depth semi-structured interviews with patients and organizational stakeholders and perform economic evaluation of site-specific implementation costs. These processes will result in a comprehensive cross-case analysis of different organizational contexts. We will utilize qualitative data analysis and configurational comparative methodology to identify facilitators and barriers at the organizational level that are minimally sufficient and necessary for optimal LS screening implementation.

**Discussion:**

The overarching goal of this project is to combine our data with theories and tools from implementation science to create an organizational toolkit to facilitate implementation of LS screening in various real-world settings. Our organizational toolkit will account for issues of complex coordination of care involving multiple stakeholders to enhance implementation, sustainability, and ongoing improvement of evidence-based LS screening programs. Successful implementation of such programs will ultimately reduce suffering of patients and their family members from preventable cancers, decrease waste in healthcare system costs, and inform strategies to facilitate the promise of precision medicine.

**Trial registration:**

N/A

**Electronic supplementary material:**

The online version of this article (10.1186/s12913-018-3636-2) contains supplementary material, which is available to authorized users.

## Background

### Rationale

The goal of precision medicine is to improve health outcomes by tailoring healthcare based on an individual’s genomic and other relevant information, including patient preferences [1]. One example is systematic screening of colorectal cancer (CRC) tumors to identify all patients whose CRC may be related to Lynch syndrome (LS) [2, 3]. LS is the most common form of inherited CRC and is also associated with significant risk for endometrial, ovarian, gastric, small bowel, and renal cancers, among others [4, 5]. LS most commonly results from the inactivation of the DNA mismatch repair (MMR) system. A first step in screening for LS in patients with cancer involves testing tumor tissue for signs of MMR deficiency in one of four genes (*MLH1, MLH2, MLH6,* and *PMS2*) [2]. Following a positive tumor screening test, additional testing for the presence of a germline pathogenic variant in one of the MMR genes is needed to confirm diagnosis of LS. A diagnosis of LS influences cancer screening and surveillance guidelines to be followed and treatment options for those already diagnosed with cancer. Patients with CRC who have LS benefit from treatment with immunotherapy [6] and have the option for more extensive colonic surgery to decrease the risk of metachronous malignancy, which is more common in LS [4, 5]. Additionally, prophylactic hysterectomy, and salpingoophorectomy can reduce risk of endometrial and ovarian cancer (90–100%) in women with LS [7].

Approximately one million people in the US have LS, of which only about 2% are aware [8, 9]. Therefore, most are not receiving potentially life-saving surveillance and treatment. In addition, at-risk family members, who are at 50% risk to also have LS, are not being identified. Cost-effective, evidence-based systematic screening strategies to identify cancer patients with LS are available and have broad recommendation for implementation [2, 10–16], yet LS screening is inconsistently applied within healthcare systems, if at all [17–19]. Systematic (also called, “universal”) screening of all newly diagnosed cases of CRC for LS is cost-effective by most measures [20, 21] and is classified by the Centers for Disease Control (CDC) as having top-tier evidence for reducing cancer morbidity and mortality and improving quality of life [11, 22]. LS screening was first recommended by the Evaluation of Genetic Application in Practice and Prevention (EGAPP) working group in 2009, and is currently recommended by multiple professional organizations, including the National Comprehensive Cancer Network (NCCN), and the Healthy People 2020 initiative [3, 10, 12–16, 23]. LS screening was also recently recommended by the Blue Ribbon Panel Precision Prevention and Early Detection Working Group to meet the goals of the Cancer Moonshot [9].

Contextual factors such as organization mission, patient population, and costs may all influence decisions to implement genomic technologies in healthcare systems [17, 24–27]. However, there is poor understanding of how these and other contextual factors impede or facilitate LS screening implementation in healthcare systems and under what circumstances [17, 18]. Factors specific to LS screening implementation may include: variable involvement of multiple key stakeholders and champions, availability of genetic counseling, genetic testing costs, and perception of value to the healthcare system [18, 19, 28]. Previous research has shown that institutional testing costs, number of CRC patients diagnosed per year, local prevalence of LS, and perceived cost of screening older cancer patients are key determinants for organizational decision makers and likely key barriers to LS screening implementation [28–30]. Additionally, there are multiple evidence-based protocols that provide different recommendations for LS screening [4, 10, 31]. These conflicting recommendations are not surprising in the face of evolving evidence, but they are likely contributors to variability in implementation. For instance, the NCCN guidelines suggest that if all CRCs cannot be tested, then testing should occur on tumors of patients diagnosed under age 70 and for patients with CRC over age 70 who meet complex criteria using family history or tumor characteristics associated with increased LS risks [4, 15]. This conflicts with evidence demonstrating that imposing age restrictions can result in substantive loss of LS index case finding, is challenging to implement, and assessment of family history and other risk factors often fails to identify individuals who are appropriate candidates for LS testing [8, 32].

However, LS screening is only successful if patients who screen positive (by tumor tissue testing) are informed and follow-through with confirmatory germline testing so that patients and their family members can access appropriate cancer treatment, screening, and prevention options. A functional tracking system and/or other processes to ensure program success is therefore an additional component of critical importance to LS screening. A study of several medical centers found that positive results were often not disclosed and poor rates of patient follow-through to germline confirmation occurred until such procedures were included in LS screening programs [19]. Finally, healthcare systems vary in implementation across a spectrum from no organized LS screening to optimal LS screening. The latter is defined as including implementation or maintenance of cost-effective tumor screening protocols, procedures to ensure disclosure of positive screening, and active processes to facilitate patient follow-through to germline genetic testing for diagnosis of LS [33–35]. An additional file shows a typical optimal LS screening program design [see Additional file 1].

### Guiding framework

Due to the complexity of LS screening and the variability of healthcare systems in which screening is to be implemented, a systematic approach based on the principles of implementation science is needed to ensure the success and sustainability of LS screening programs. The Consolidated Framework for Implementation Research (CFIR) has been used successfully to evaluate variation in program implementation [36, 37] and to identify conditions necessary for patient follow-through to confirmatory gene sequencing in LS screening programs [19]. The CFIR describes several processes that may influence implementation success and guides assessment of contextual conditions that may serve as implementation barriers or facilitators at the individual, organizational, and external levels. The CFIR also guides data gathering and structuring for conducting configurational comparative analysis to identify which CFIR constructs and LS screening program-specific procedures are minimally sufficient and necessary for optimal implementation, and under which circumstances in complex health care delivery systems [19, 38, 39].

### Objectives and aims

The goal of this project is to utilize the CFIR and other tools from implementation science to describe, compare, and explain variations in LS screening across multiple healthcare systems and create a comprehensive, customizable organizational toolkit for implementing and improving LS screening programs. To achieve this goal, this study has four specific aims:

**Aim 1:** Describe variations in LS screening, implementation processes, and contextual conditions facilitating or impeding implementation across multiple healthcare systems.

**Aim 2:** Explain current practice variation and identify contextual conditions that influence variation in LS screening, including follow-up procedures, and implementation processes to identify which combinations are minimally sufficient and necessary for optimal implementation.

**Aim 3:** Determine the relative effectiveness and costs of different LS screening protocols using decision analytic models developed from previous work [29, 30] and data specific to each healthcare system to demonstrate the relative effectiveness and efficiency of various LS screening protocols used by healthcare systems based on their local data.

**Aim 4:** Develop an organizational toolkit to facilitate LS screening implementation and optimization and disseminate to all sites. Further assess the toolkit’s utility for facilitating LS screening implementation or optimization then refine and for broader dissemination.

## Methods/design

### Overall study design

This study combines multiple methods of exploring implementation in complex healthcare systems to determine which implementation strategies are likely to work best in different organizational contexts. We will use traditional case-based, in-depth analyses of conditions that may serve as barriers and facilitators of LS screening (Aim 1), followed by cross-case and configurational comparative analysis to determine minimally sufficient and necessary conditions for optimal LS screening (Aim 2). These data will be used to populate economic evaluation decision analytic models to demonstrate how organizational context, available resources, and screening protocol impact organizational costs and program success (Aim 3). A toolkit will be created and disseminated to sites to guide implementation of new programs that are aligned with the organizational context and costs or to optimize existing programs (Aim 4).

### Study setting

The unit of analysis is the clinical site through which LS screening is or can be implemented. We will study the contextual factors of 21 clinical sites across 7 healthcare systems purposively selected to maximize the number of clinical sites in various stages of implementing LS screening, as well as to maximize diversity of location, system structures, and patient populations (Table 1). Six healthcare systems (Kaiser Permanente Northwest and Colorado, HealthPartners, Harvard Pilgrim Healthcare, Meyers Primary Care Institute/Reliant Medical Group, Sutter Health-Palo Alto Medical Research Foundation, and Geisinger) are part of the Healthcare Systems Research Network (HCSRN), and one is a national non-profit faith-based system with locations in 18 states (Catholic Health Initiatives). Of the six HCSRN sites, Kaiser Permanente sites are integrated healthcare systems where patients are members, while the other sites are “open” systems where patients can be members, patients, or patient members (e.g. members receiving care in the system or outside the system, or patients receiving care who are not members of the health plan).

### Data collection aims 1 and 2

A comprehensive case description of each organization, including practice variation and factors influencing implementation, evaluation, maintenance, and improvement of LS screening, will be created through qualitative interviews of organizational stakeholders and patients with newly diagnosed CRC. For sites with LS screening programs, additional interviews will be conducted with patients diagnosed with LS. Interviews of organizational stakeholders will be conducted centrally by trained staff at Geisinger. Interviews of patients will be conducted centrally by trained staff at Kaiser Permanente Northwest (KPNW). Consent will be obtained at the start of the interview process and patient interviewees will receive a $25 gift card upon completion of the interview.

#### Organizational stakeholder recruitment

Up to 10 organizational stakeholders per site will be recruited through purposive role-based recruitment and snowball sampling [40, 41]. The actual number of and specific key stakeholders interviewed will depend on the site’s organizational structure. Typical stakeholders relevant to LS screening include: pathologists, genetic counselors, gastroenterologists, oncologists, surgeons, healthcare administrators, and health plan leaders. Research staff from each site will reach out to initial stakeholders from their organization via email or other methods to alert them to the study and invite them to participate in a telephone interview. At the end of each completed interview, the interviewee will be asked to identify any additional organizational stakeholders necessary for implementing new processes generally and LS screening specifically (snowball sampling). Additional stakeholders will be sent an email indicating that they were nominated to be invited into the study and offered the opportunity to participate in a telephone interview.

#### Patient stakeholder recruitment

Consideration of patient needs is another CFIR construct that may impact implementation success; therefore, it is critical to gather their feedback about LS screening. Patient follow-through with germline confirmation of LS is required for tumor screening to impact downstream clinical care processes. Likewise, anticipation of adverse patient reactions by organizational decision-makers can be a barrier to implementation at the system level. Two different groups of patient stakeholders will be invited to participate in this study: [1] patients newly diagnosed with CRC (*N* = 10 per site) and [2] patients who have been notified of a positive LS screen result and were recommended for additional genetic counseling and testing to confirm a diagnosis (*N* = 25 total across all sites with LS screening). For patients with newly diagnosed CRC, study staff at each site will determine the best way to identify and contact patients 3–6 months post-diagnosis and offer the opportunity to participate in this one-time telephone interview. This group will illuminate local patient attitudes and opinions about LS screening for organizational decision-makers, while the diversity of these patients across all sites will provide insight into patient attitudes in general towards LS screening. Patients who have received a positive LS screen result will provide insight into patient experiences with a positive LS screen across different sites and different LS screening protocols.

#### Qualitative data collection

Based on prior work [19, 40–42], it is anticipated that most organizational stakeholders will participate in interviews, resulting in sufficient information from each site to create a comprehensive case description with consistent detail for comparison across sites. While the number of patients interviewed per site is small (*n* = 10), the total number of patients across all sites (*n* = 220) is large for qualitative research and will result in sufficient sample size to capture the range of experiences, expectations, and preferences of newly diagnosed cancer patients from diverse backgrounds and healthcare systems related to LS screening.

As used previously by this research team and others, semi-structured interviews will be conducted via telephone centrally as noted above by staff experienced in qualitative data collection [40–44]. A summary will be created immediately after each interview and reviewed with site investigators during regular study meetings. These summaries will be used to modify the sampling procedure or interview guides if needed, to create the initial coding schema, and to create the comprehensive case description of each organization. Such summaries allow for high-level analysis during on-going data collection, facilitate codebook development, and reduce the number of de novo codes requiring re-review and re-coding of transcripts during data analysis [45, 46].

The CFIR-guided constructs to be assessed through the organizational and patient stakeholder interview guides are detailed in Table 2. The organizational stakeholder interview guide has been developed and refined using the question bank available from the CFIR website [47] and will be further tailored to the position of the key stakeholder. For example, system leaders may be asked more questions about engagement of leadership, external influences, and reimbursement incentives [40]. The patient interview guide has been adapted from a prior study [44].

### Data analyses aims 1 and 2

We will use CFIR and the case summaries created for each site to guide cross-case comparisons with the purpose of describing contextual variation associated with where, when, and under which conditions different processes for implementing or improving LS screening might be successful [47]. The result will be a descriptive summary of patterns in variations across sites, which will provide the basis for selecting conditions for inclusion in the analyses for Aim 2.

**Fig. 1:**
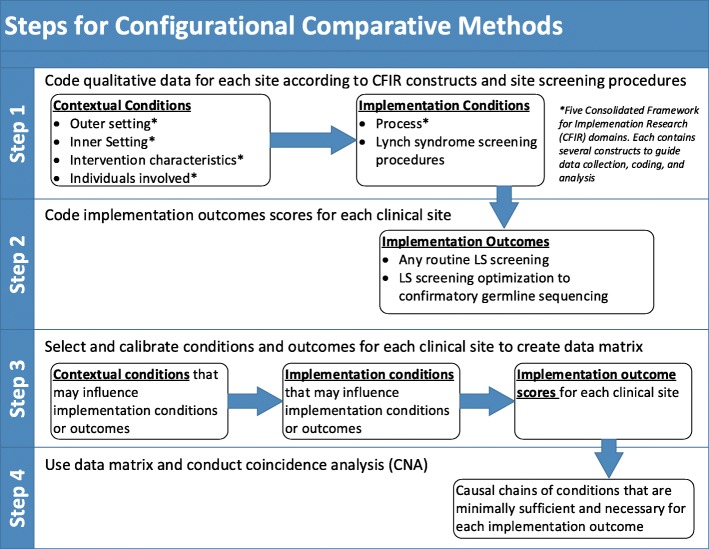
Configurational Comparative Method Conceptual Model and Analytic Approach

Configurational comparative methods (CCM), such as Qualitative Comparative Analysis (QCA) and coincidence analysis (CNA) are particularly suitable analytic techniques for studying causal complexity in organizational implementation (e.g. multiple conditions may combine in various ways to cause the same outcome) [19, 38, 48–50]. CNA was selected for use in our study because, unlike QCA, it uses a different minimization algorithm that can identify underlying causal chains [51]. This is useful because we anticipate the presence or absence of some contextual conditions may impact one or more combinations of other contextual or implementation conditions that are minimally sufficient and necessary for the outcome under investigation. Data analyses for Aim 2 will consist of 4 main steps [38]: 1) code data for context and implementation conditions (CFIR constructs), 2) code implementation outcomes, 3) calibrate conditions and outcomes to create data matrix and 4) conduct CNA and interpret solutions to create a model of minimally sufficient and necessary conditions for defined outcomes (Fig. 1).

#### Coding LS screening contextual and implementation conditions (step 1)

All patient and organizational stakeholder interviews will be digitally recorded and transcribed verbatim. Transcripts will be uploaded into Atlas.ti (www.atlasti.com) for qualitative analysis. Transcripts will be initially coded using an a priori codebook developed from the semi-structured interview guides, interview summaries, and CFIR constructs. This first round of coding (Aim 1) will look for description of LS screening procedures, process of implementing LS screening, individuals involved and inner or outer setting as shown in Table 2. Emergent (de novo*)* codes will be added to any other relevant sections of transcript text not fitting the a priori codes. This coding will be an iterative process involving team members independently coding 2–3 transcripts at a time, then discussing their coding to adjust the codebook and subsequently to group codes into categories or themes. This process will continue until the code list is static, all transcripts are coded, and the categories and themes are finalized. Additional codes will note whether contextual conditions were reported to impact the site’s implementation processes or procedures and how. A minimum of two researchers will independently code and resolve disputes by consensus until all conditions and impact are coded. Coding will follow protocols detailed in the CFIR technical assistance website [47] .

#### Coding implementation outcomes (step 2)

Two implementation outcomes will be examined: [1] implementation of any type of LS screening and [2] optimal LS screening implementation. As noted above, conditions for optimal implementation include: implementation or maintenance of any systematic LS screening without limitations; quality tracking to confirm testing of all tumors and disclosure of all positive screens, and systems for facilitating patient follow-through with confirmatory gene sequencing. An additional file (see Additional file 2) shows how the domains and constructs of the CFIR will be utilized to code the stakeholder information from each site.

Sites conducting any tumor screening on all or a clearly defined subset of endometrial and/or colorectal tumors will be scored 1 and those that have not yet implemented routine screening will be scored 0 for the first implementation outcome (Sites with no program in Table 1 vs. sites with any type of program). Using data from interview transcripts, site summaries, and the ability of sites to easily access numbers of patients screened, proportion who screen positive and proportion of those who follow-through with confirmatory testing and counseling. At least two members of the research team will independently code the second implementation outcome on a scale of 0 (tumor testing with no follow-up procedures) to 5 (fully-functioning tumor screening with facilitation and confirmation of germline testing). Coding conflicts will be resolved as described previously.

**Table 1:** Participating Healthcare Systems and Lynch Syndrome Screening by Clinical Site

		Lynch Syndrome Screening Program Type^a^					
			CRC Tumor Screening^b^			EC Tumor Screening^c^	
Healthcare System	Clinical Site (State)	No Program	CRC age cutoff	BRAF Reflex	PHM Reflex	EC age cutoff	PHM Reflex
Geisinger	Geisinger (PA)		All ages	X	X	All ages	X
Kaiser Permanente (KP)	KP-Colorado (CO)	X					
	KP-Northwest (OR/WA)		All ages	X	X	All ages	X
Sutter Health (SH)	SH-Palo Alto Medical Foundation (CA)	X					
Meyers Primary Care Institute (MCPI)	Reliant Medical Group (MA)		All ages				
HealthPartners	HealthPartners (MN)		All ages	X	X	All ages	X
Harvard Pilgrim Healthcare	Harvard Pilgrim (MA)	X					
Catholic Health Initiatives (CHI)	Franciscan (WA)		All ages	X	X	All ages	X
	Tri Health (OH)		All ages	X	X	All ages	X
	Mercy Des Moines (IA)		All ages	X	X	All ages	
	Kentucky One (KY)		All ages				
	Chattanooga (TN)		All ages	X	X	All ages	X
	Good Samaritan (NE)	X					
	Lincoln (NE)		< 70 years	X			
	St. Francis (NE)	X					
	St. Joes Bryan (TX)	X					
	St. Vincent (AR)	X					
	Centura (CO)		< 70 years			< 60 years	
	Alegent Creighton (OH)	X					
	St. Alexius (ND)	X					
	Mercy (ND)	X					

^a^Recommendation: screen all Colorectal cancer (CRC) and Endometrial Cancer (EC) tumors for mismatch repair deficiency

^b^Recommendation: reflex test positive screens for *BRAF*V600E point mutation and MLH1 Promoter Hypermethylation (PHM) testing to rule out somatic loss of function (sporadic cancer)

^c^Recommendation: reflex test positive screens for PHM to rule out somatic loss of function (sporadic cancer)

**Table 2:** CFIR Constructs by Domain Specific to LS Screening to be Assessed in Stakeholder Interviews

CFIR Domain	CFIR Constructs Specific to LS Screening
Intervention Characteristics	Adaptability of LS screening to local context
	Perceived difficulty implementing LS screening
	Cost to the organization associated with screening
Outer Setting	Patient needs and resources
	Competitive pressure to implement screening
	Impact of external policies on organization
Inner Setting	Organization structure
	Perceived organizational priority to implement
	Implementation climate in organization
Characteristics of Individuals	LS knowledge and beliefs, perceptions of evidence
	Individual readiness to implement screening
	Self-efficacy to complete actions in screening
Implementation Process	Planning process to implement LS screening
	Champions, opinion leaders, and other stakeholders
	Tracking and feedback processes for LS screening

#### Selecting and calibrating conditions and outcomes to create a data matrix (step 3)

Following standard practices for CCM, we will select which conditions to include using theoretical and empirical knowledge of the cases [52–54]. To complete this task at least two members of the research team will review the coded data and select a set of approximately 4 to 8 conditions hypothesized to impact each outcome. Final consensus will be reached through discussion among coders and other research team members who have substantive experience with LS screening. Our second implementation outcome and any non-binary conditions will systematically be calibrated using theoretical knowledge and recommended practices in order to indicate the degree to which the outcome or condition is present or absent [52, 54]. The calibrated conditions and outcomes for each site will be compiled into a numerical data matrix for use in conducting coincidence analysis (CNA).

#### Conducting coincidence analyses (step 4)

Coincidence analyses (CNA) will be conducted following standard procedures [51–54] using the CNA package in R (www.R-project.org) with the main goal of identifying causal chains of conditions that are minimally sufficient and necessary for each of the two main implementation outcomes [55]. Additional CNA will be conducted to identify which contextual conditions (if any) contribute to variability in implementation processes or procedures that are hypothesized or have previously been shown to be more efficient, cost-effective, or result in better patient ascertainment and completion of confirmatory genetic testing [19, 29, 30, 33–35].

The resulting CNA solutions will be interpreted by the research team to identify implications for optimal LS screening implementation. This will be combined with the economic analysis data from Aim 3 to form the basis for the toolkit to help organizational decision makers determine what implementation strategies are more likely to work in optimizing screening given their organizational context.

### Procedures aim 3

In Aim 3 we will calculate expected outcomes central to evaluation of effectiveness and efficiency of LS screening in CRC and endometrial cancer (EC) patient populations. Previously developed models [29, 30] will be modified and updated based on recent developments, current evidence and guidelines. Models will be populated using local data to reflect site-specific conditions as determined from data collected from study site stakeholder interviews in Aim 1.

We will construct decision tree-type analytic models in Excel© software (Microsoft, Inc., Redmond, Wash.), with the @Risk© software add-on for Excel (Palisade, Inc., Ithaca, NY) to enable various complex analytic procedures. The decision analytic team will construct multiple decision tree models to represent all LS screening protocols the team considers currently viable. Model variables include estimates of annual incident CRC and EC cases by age strata, LS prevalence or the assumption of an equivalent rate for all populations, and cost of tests included in screening protocols from each site to populate decision analytic models. When institutional cost is not available, alternative methods such as the average test cost based on costs reported by other participating clinical sites, regional test cost figures if publicly available from testing companies, or Medicare reimbursement rates will be used. Finally, reliable estimates of site-specific LS prevalence may not be available; therefore, this model parameter may be estimated from the most current estimates for U.S. populations [56]. Because clinical sites, and scientific evidence are dynamic, we do not expect organizational resources, testing costs, or LS screening guidelines to stay static. Therefore, the decision analytic models developed will be designed in such a way to facilitate ongoing use and modification by organizations.

### Analyses aim 3

For base-case and sensitivity analyses, the decision analytic models will calculate, for each site and in CRC and EC virtual cohorts (e.g. 500 cases per year): (1) sensitivity and specificity of each LS screening protocol in identifying LS cases including number of LS cases expected to be identified, (2) total costs for each screening protocol, (3) cost per CRC and EC case-screened, (4) cost-per LS index case identified, (5) incremental differences between guideline-based screening protocols including costs and LS cases identified, (6) and patient adherence to each screening protocol including number of CRC and EC cases lost to follow up. Additional modeling of different LS screening age cut-off policies using local-level data will be conducted for each site. Models will provide objective metrics, driven by local data, of the impact of applying age-cutoffs in LS screening implementation.

### Procedures aim 4

Data from Aims 1, 2, and 3 will be used to generate a working organizational toolkit to guide implementation, maintenance, and optimization of LS screening. Additional information will be collected over the entire project period from monthly project meetings, communications from site investigators, pertinent data regarding site-specific screening changes, and external evidence or guideline changes for LS screening. These data will be recorded in a project specific database created for tracking such information related to implementation [57]. This tracking database will provide important information for the toolkit development should any sites begin to implement LS screening based on being interviewed for Aim 1, but prior to receiving the toolkit.

The organizational toolkit will be created based on the CFIR conceptual framework, the in-depth knowledge of LS screening programs and contextual factors of healthcare systems from Aim 1, the cross-site comparison and CNA results from Aim 2, and economic evaluation by decision analytic modeling with local data from Aim 3. This toolkit will be disseminated to all sites through site investigators and the tracking database will record to whom it is distributed, questions asked by those receiving the toolkit, and any immediate actions taken by the site.

Six months after distribution of the toolkit, additional qualitative interviews will be conducted with up to 5 organizational stakeholders at each site using the same processes described for Aim 1. Stakeholders from sites without LS screening and those with sub-optimal implementation will be interviewed about the utility of the tool to facilitate implementation and improvement. Stakeholders from sites with optimally implemented programs will be interviewed about the utility of the tool for improvement or adaptation to emerging evidence. The interview guide for this aim will be developed by the research team in parallel with the development of the toolkit.

### Analyses aim 4

Interviews will be conducted, transcribed, and coded as described for Aim 1. Interviews will be coded for information on to whom the organizational toolkit was distributed at each site, questions that were asked by key stakeholders, and whether and how the toolkit was used by organizational decision makers to facilitate LS screening implementation and/or improvement.

## Discussion

### Dissemination

The final organizational toolkit for LS screening implementation, maintenance, and improvement will be modified based on the information learned throughout the study and from the pilot distribution to all study sites. The final product will include descriptions of most commonly included stakeholders and general processes needed for optimal LS screening, directions for processes and protocols that are more likely to work by contextual factors identified, and a generic decision analytic model for costs and effectiveness related to available LS screening protocols. This organizational toolkit will have an intuitive interface that allows for the input of local parameters for use by organizational decision-makers. This toolkit will be distributed through the Lynch Syndrome Screening Network (LSSN; www.lynchscreening.net) and through other channels that may be identified.

### Innovation

Through in-depth assessment of contextual conditions impacting LS screening implementation across an unprecedented number of sites representing diverse healthcare systems, geographies, and patient populations served, this study will provide significant information for the Precision Prevention and Early Detection Working Group of the Blue Ribbon Panel to successfully address the Cancer Moonshot [9], and for the working group of National Academies of Sciences, Engineering, and Medicine (NASEM) to successfully facilitate their goal of broadly implementing LS screening [58]. This study will also contribute to implementation science more generally, as it combines multiple methods to extend beyond the typical “lessons learned” approach and utilizes a relatively new analytic approach to illuminate minimally necessary and sufficient conditions for LS screening implementation in different organizational contexts. Another innovation of this study is our ability to provider tailored information to each site by combining key stakeholder information with business case decision analytic models that can be populated with local, real-world data. The relevance of general societal cost to organizational decision-making has been questioned [59, 60] and prior studies indicate that organization-specific costs to screen and to detect LS cases for different protocols is critical information for health systems to make decisions about LS screening implementation [28–30].

To our knowledge, no studies have synthesized in-depth cross-site comparison of context, barriers and facilitators with local business case analyses into a comprehensive toolkit for organizations implementing and optimizing LS screening programs. Most studies to date have focused on strategies for initial implementation, rather than maintenance and improvement in the face of organizational context or changes in evidence. This study will fill that gap and provide insights into successful implementation in the era of precision medicine where evidence is evolving and costs and opportunities for genomic testing are rapidly changing.

### Limitations and challenges

Healthcare systems and evidence are dynamic; therefore, it is possible that any of the participating sites may implement LS screening during the course of the study, or that sites currently implementing LS screening may optimize their programs prior to receiving the toolkit. This possibility may decrease the diversity of our sites in contextual or implementation conditions or outcomes; thus, changes will be tracked to ensure the integrity of the analyses. Additionally, new evidence [61] and declining costs of sequencing may lead to the replacement of currently recommended LS screening protocols with tumor sequencing or even germline sequencing of all newly diagnosed cancer patients. Because economic evaluation is an integral part of this study and direct sequencing cost comparison will be included in the organizational decision-making guide, this changing evidence can easily be re-evaluated by each organization’s decision-makers over time. Because this study is specifically designed to collect cost and contextual information throughout the course of the study, we will utilize changes made at any site over the course of the study as data to inform implementation, maintenance, and adaptation broadly. Therefore, this study will not only result in a toolkit to guide LS screening implementation in an era of dynamic and changing evidence and costs, but may also broadly demonstrate for the field of implementation science methods for conducting research in the real-world that are focused on optimization, maintenance, and adaptation to new evidence.

### Summary and impact

This study addresses a major unmet need identified by the Blue Ribbon Panel to achieve the goals of the Cancer Moonshot and to improve our understanding of clinical implementation of complex interventions [9]. The overarching goal of this study is to determine factors associated with variations in LS screening implementation across multiple healthcare systems and to provide the organizational decision tools, or “recipes,” for implementation success in light of different organizational contexts and costs. The organizational toolkit that we produce will help health systems to maintain and optimize LS screening programs in the face of changing costs and evidence. Our toolkit based on implementation science may be adapted to help in implementing evidence-based screening for other genomic conditions or for implementing other types of complex genomics interventions into healthcare systems.

## Additional Files

Additional file 1: Suggested Optimal Lynch Syndrome Screening Program Protocol. This file shows the flow diagram of the suggested optimal design for a LS screening program protocol based on current guidelines. (PDF 78 kb)

Additional file 2: Analytic Model Showing CFIR Constructs by Domain for Coding. This file diagrams how the key stakeholder interview data will be coded by CFIR constructs and domains, and how we will analyze across cases to determine barriers and facilitators of LS program development. (PDF 104 kb)

## Abbreviations

CDC: Centers for Disease Control
CFIR: Consolidated Framework for Implementation Research
CHI: Catholic Health Initiatives
CNA: Coincidence Analysis
CRC: Colorectal cancer
EGAPP: Evaluation of Genetic Application in Practice and Prevention
KP: Kaiser Permanente
KPCO: Kaiser Permanente Colorado
KPNW: Kaiser Permanente Northwest
LS: Lynch syndrome
MPCI: Myers Primary Care Institute
NASEM: National Academies of Science, Engineering, Medicine
NCCN: National Comprehensive Cancer Network
PAMFRI: Palo Alto Medical Research Foundation Institute
QCA: Qualitative Comparative Analysis
