# Supplementary material for: Implementing universal Lynch syndrome screening (IMPULSS): protocol for a multi-site study to identify strategies to implement, adapt, and sustain genomic medicine programs in different organizational contexts
Source: BMC Health Serv Res. 2018 Oct 30;18:824. doi: 10.1186/s12913-018-3636-2 (PMC6208012; doi:10.1186/s12913-018-3636-2)
Supplement: Supplementary file 1 — Suggested Optimal Lynch Syndrome Screening Program Protocol. This file shows the flow diagram of the suggested optimal design for a LS screening program protocol based on current guidelines. (PDF 78 kb) [file 12913_2018_3636_MOESM1_ESM.pdf]

Optimal Lynch Syndrome Screening Program Protocol

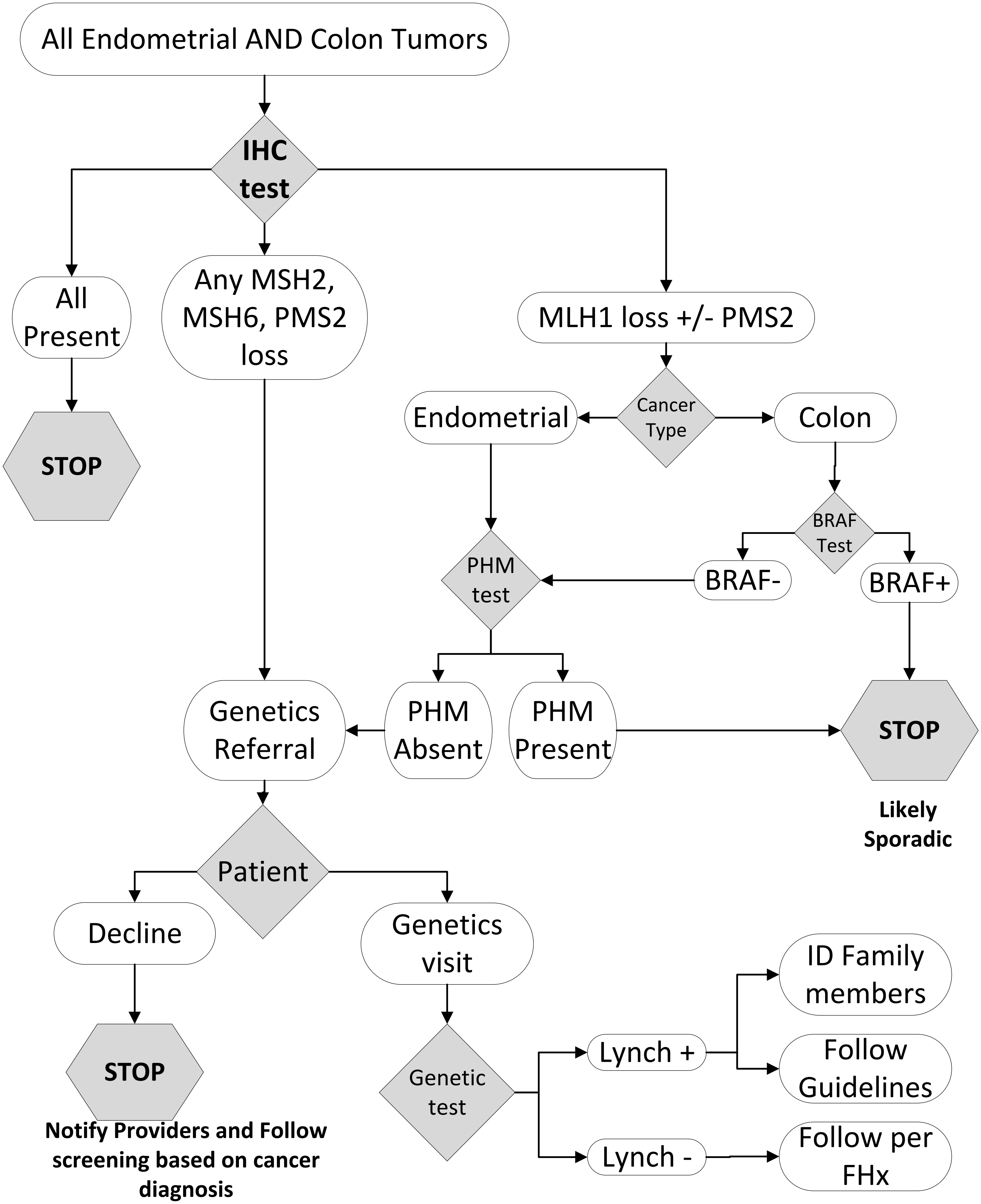

PHM: Promoter hypermethylation; IHC: Immunohistochemistry; FHx: Family History
