# Supplementary figures and images for: Implementing universal Lynch syndrome screening (IMPULSS): protocol for a multi-site study to identify strategies to implement, adapt, and sustain genomic medicine programs in different organizational contexts
Source: BMC Health Serv Res. 2018 Oct 30;18:824. doi: 10.1186/s12913-018-3636-2 (PMC6208012; doi:10.1186/s12913-018-3636-2)

# Analytic Model Showing CFIR Constructs by Domain For Coding

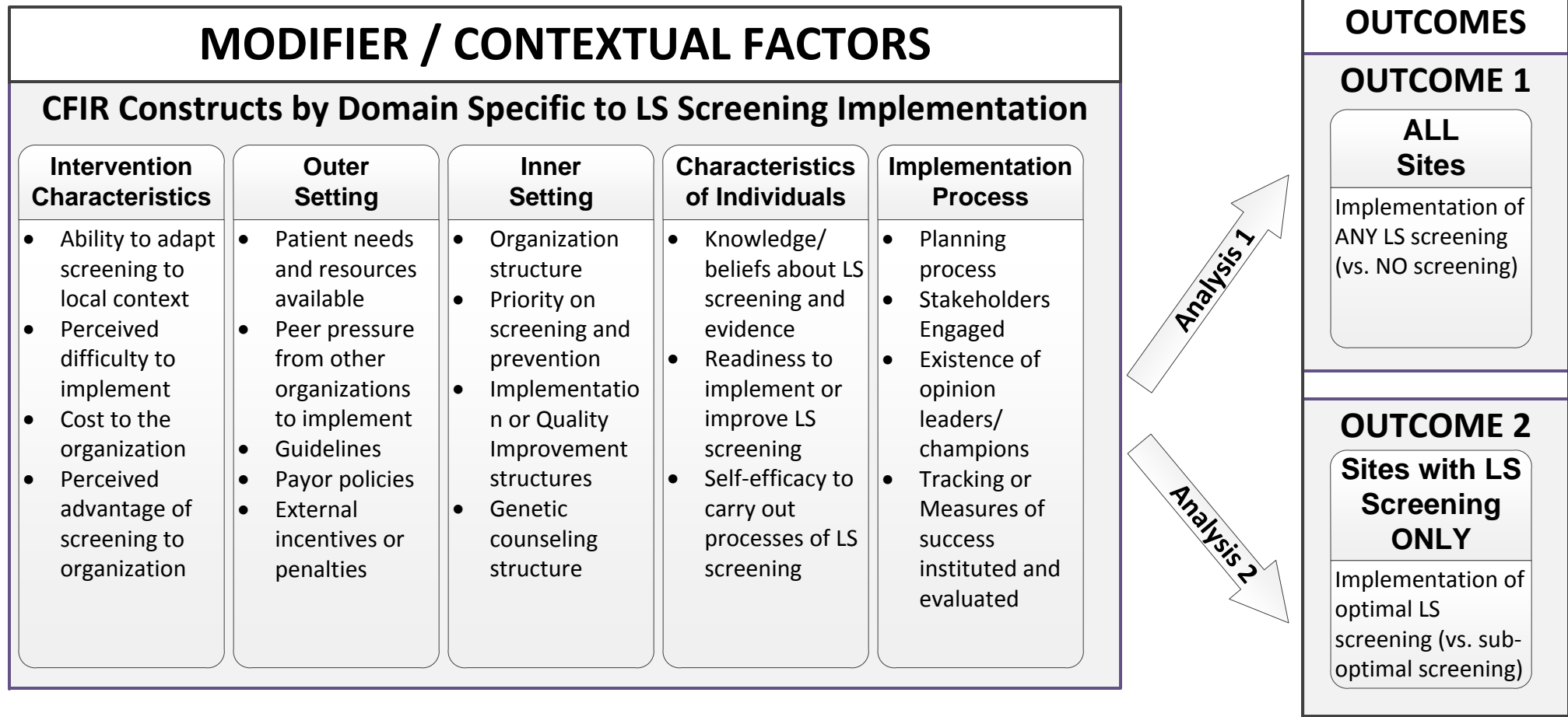

Supplement: Supplementary file 2 — Analytic Model Showing CFIR Constructs by Domain for Coding. This file diagrams how the key stakeholder interview data will be coded by CFIR constructs and domains, and how we will analyze across cases to determine barriers and facilitators of LS program development. (PDF 104 kb) [file 12913_2018_3636_MOESM2_ESM.pdf]
